# Systems-level barriers to treatment in a cervical cancer prevention program in Kenya: Several observational studies

**DOI:** 10.1371/journal.pone.0235264

**Published:** 2020-07-13

**Authors:** Charlotte M. Page, Saduma Ibrahim, Lawrence P. Park, Megan J. Huchko

**Affiliations:** 1 Department of Obstetrics and Gynecology, Duke University, Durham, North Carolina, United States of America; 2 Kenya Medical Research Institute, Nairobi, Kenya; 3 Division of Infectious Diseases, Department of Medicine, Duke University, Durham, North Carolina, United States of America; 4 Duke Global Health Institute, Duke University, Durham, North Carolina, United States of America; Sapienza University of Rome, ITALY

## Abstract

**Objective:**

To identify health systems-level barriers to treatment for women who screened positive for high-risk human papillomavirus (hrHPV) in a cervical cancer prevention program in Kenya.

**Methods:**

In a trial of implementation strategies for hrHPV-based cervical cancer screening in western Kenya in 2018–2019, women underwent hrHPV testing offered through community health campaigns, and women who tested positive were referred to government health facilities for cryotherapy. The current analysis draws on treatment data from this trial, as well as two observational studies that were conducted: 1) periodic assessments of the treatment sites to ascertain availability of resources for treatment and 2) surveys with treatment providers to elicit their views on barriers to care. Bivariate analyses were performed for the site assessment data, and the provider survey data were analyzed descriptively.

**Results:**

Seventeen site assessments were performed across three treatment sites. All three sites reported instances of supply stockouts, two sites reported treatment delays due to lack of supplies, and two sites reported treatment delays due to provider factors. Of the 16 providers surveyed, ten (67%) perceived lack of knowledge of HPV and cervical cancer as the main barrier in women’s decision to get treated, and seven (47%) perceived financial barriers for transportation and childcare as the main barrier to accessing treatment. Eight (50%) endorsed that providing treatment free of cost was the greatest facilitator of treatment.

**Conclusion:**

Patient education and financial support to reach treatment are potential areas for intervention to increase rates of hrHPV+ women presenting for treatment. It is also essential to eliminate barriers that prevent treatment of women who present, including ensuring adequate supplies and staff for treatment.

## Introduction

In East Africa, cervical cancer is the most common cancer among women. This region has the highest incidence of and mortality from cervical cancer in the world; age-adjusted mortality was estimated at 16 in 100,000 in East Africa in 2018 compared to 1 per 100,000 in North America [1]. Cervical cancer prevention begins with screening, and in Kenya, screening coverage is only 3.5% [2]. Screening alternatives to cytology are recommended for low-resource settings, and the most effective of these at reducing cervical cancer mortality is testing for high-risk human papillomavirus (hrHPV) [3, 4]. To be effective, hrHPV testing must be part of a cervical cancer prevention cascade that includes education, screening, communication of results, and linkage to treatment.

As hrHPV testing is relatively new in low- and middle-income countries such as Kenya, there is limited data on barriers to women’s completion of a cervical cancer prevention cascade after screening. Barriers may arise at the steps of deciding to get treated, navigating the treatment process, and receiving treatment. Research by Geng et al on loss-to-follow-up among HIV patients in East Africa has identified structural barriers (e.g. transportation), clinic-based barriers (e.g. wait time), and psychosocial barriers (e.g. stigma) as contributing factors [5, 6]. We have previously reported on patient factors associated with whether women who screened hrHPV+ presented for treatment [7]. The aim of the current research was to identify health systems-level barriers to treatment, including both structural and clinic-based barriers.

## Materials and methods

The current research was nested within a two-phase cluster-randomized trial of implementation strategies for cervical cancer prevention in Migori County in western Kenya (registered at ClinicalTrials.gov, identifier NCT02124252, https://clinicaltrials.gov/ct2/show/NCT02124252?term=NCT02124252&rank=1; protocol available at https://dx.doi.org/10.17504/protocols.io.6s5heg6) [8]. Recommendations by the World Health Organization were the basis for the screening protocol, and the implementation strategies were informed by previous work in the region [9]. It was the first protocol in Kenya to incorporate hrHPV testing as part of screening through government health facilities.

Screening for hrHPV with self-collected specimens was offered at community health campaigns (CHCs) to women in Migori County who were eligible for cervical cancer screening based on the Kenya Ministry of Health’s guidelines, i.e. women aged 25–65 years [10]. CHCs were conducted sequentially in six rural communities in Migori County, each operating for two weeks between February and October 2018. The communities were Lwanda, Olasi, Kituka, Kabuto, Osingo, and Ogwedhi. In the weeks preceding the CHCs, study staff met with community leaders and used poster, leaflet, and radio advertising to publicize the dates and activities of the CHCs. In order to reach the entire community, each campaign moved to multiple sites over its two-week period, with approximately four days at each site. Given this recruitment strategy, the women who registered at the CHCs and enrolled in the study can be considered representative of the women in the six target communities. At the campaigns, women self-swabbed for hrHPV after receiving education about HPV and cervical cancer. Women were notified of their hrHPV results and given follow-up instructions by either text message, phone call, or home visit, according to their preference.

Women who tested positive for hrHPV were referred for treatment at one of four government health facilities–Macalder Sub-county Hospital, Migori County Referral Hospital, Karungu Sub-county Hospital, or Ogwedhi Health Center–based on proximity to their community. Treatment was offered for each community starting two weeks after its CHC. Since data collection concluded on February 14, 2019, the treatment periods varied in length from 51 weeks for the first community to 21 weeks for the last community. Unless contraindicated by pregnancy, menses, or cervical exam, women were treated by a clinical officer or nurse with cryotherapy, which is an effective, low-cost treatment modality well-suited to low-resource settings [11]. Women with cervical lesions not amenable to cryotherapy or suspicious for cancer were referred to a gynecologist at Migori County Referral Hospital.

Women who presented for treatment completed a questionnaire prior to final eligibility for treatment that day (see S1 File for questionnaire). Questionnaires were verbally administered by research assistants, community health volunteers, nurses, or clinical officers. For the current research, the data that were used from patient questionnaires regarded wait time and roundtrip transportation costs. Wait time was defined as the amount of time from patient-reported arrival at the facility until provider-recorded intake time. These variables, along with treatment rates, were compared across the four treatment sites using Kruskal-Wallis rank tests. All data from patient questionnaires, site assessments, and provider surveys were entered directly into tablets using ODK Collect (docs.opendatakit.org). All statistical analyses were performed using STATA/SE 16.0, and a two-sided p-value <0.05 was considered significant.

During each site’s treatment period, site assessments were performed periodically, with a goal of every two weeks (see S2 File for assessment tool). The questions were posed verbally by a research assistant to a healthcare provider involved with the study, who was not necessarily the same person across all assessments at a given site. An expanded set of questions was asked at the first (baseline) assessment at each site, and an abbreviated set assessing factors that could change over time was asked at subsequent assessments. The cryotherapy supplies inventoried at each site assessment consisted of gloves, acetic acid, specula, lights, cotton swabs, mackintoshes (non-disposable plastic sheets), cryoprobes, and gas. In addition, the healthcare provider was queried as to whether treatment had been delayed in the last week due to lack of any of the abovementioned supplies. The percentage of expected providers present was the number of clinical officers and nurses present divided by the number expected that day. Site assessment variables were compared across the three treatment sites that had at least two site assessments (the fourth, Ogwedhi Health Center, was excluded because it had only one site assessment). Categorical variables were compared using Fisher’s exact tests, and numerical variables were compared using Kruskal-Wallis rank tests.

Surveys were conducted with providers from the treatment sites who were involved in counseling and/or providing treatment for hrHPV+ women (see S3 File for survey tool). All providers who could be identified were approached and asked to participate. Those who provided informed consent were surveyed verbally by a research assistant at least two weeks after treatment started at their site. Survey questions were in yes/no or multiple-choice format, and data were entered directly into tablets. In order to capture comments that providers made besides their responses to questions, the survey sessions were also audio-recorded on the tablets.

No power calculations were performed. For the participant analysis, a power calculation was not performed since we were working with data from a larger study. For the site assessments, power calculations were not performed because there were so many variables involved. Finally, for the provider surveys, power calculations were not performed since the data were only analyzed descriptively.

This study was approved by the Duke Institutional Review Board (protocol # Pro00077442) and the Scientific and Ethics Review Unit of the Kenya Medical Research Institute (protocol # 2918). Women participating in the hrHPV screening and treatment protocol provided written informed consent at the time of screening and verbal affirmation at each follow-up encounter. For women with lower literacy levels, consent was confirmed with a fingerprint. Informed consent was not necessary for the site assessments since they were considered quality improvement initiatives. Providers who participated in surveys provided written informed consent.

## Results

The flow diagram for the patient component of this study has been published elsewhere [7] and is included here as a supporting file (S1 Fig). Demographic information on the participants can also be found in a supporting file (S1 Table). Of 505 hrHPV+ women notified of their results, 266 (53%) presented for treatment. Of those who presented for treatment, 236 (89%) were treated: 229 (97%) at their first visit and seven (3%) at their second visit. Thirty women (11%) of the 266 who presented were not treated for the following reasons: 15 (6%) due to gas outage, six (2%) due to pregnancy, five (2%) due to concern for cervical cancer, and four (2%) due to an unknown or other reason.

The percentage of notified hrHPV+ women in the catchment area who were treated varied significantly across the treatment sites (Table 1), ranging from 39% to 61% (p = 0.03). The sites did not differ significantly in rates of women presenting from within their catchment area. The site with the highest treatment rate, Migori County Referral Hospital, had the lowest median transportation cost reported by participants (1.20 USD compared to 3.00 USD for the most expensive site) and the second shortest median wait time (56 minutes compared to 116 minutes for the site with the longest wait).

**Table 1:** Treatment statistics across four treatment sites in Migori County, Kenya.

Characteristic	Overall	Macalder Sub-county Hospital	Ogwedhi Health Center	Migori County Referral Hospital	Karungu Sub-county Hospital	p-value
Treatment period, in weeks		51	36	30	21	
Notified hrHPV+ women (N)	505	224	59	72	150	
Women who presented (% of notified hrHPV+ women)	266 (53%)	114 (51%)	26 (44%)	47 (65%)	79 (53%)	0.09
Women who got treated (% of notified hrHPV+ women)	236 (47%)	96 (43%)	23 (39%)	44 (61%)	73 (49%)	0.03*
Median wait time, in minutes (IQR)	67(17–143)	68 (16–124)	41 (5–124)	56 (30–120)	116 (34–180)	0.04*
Median transportation cost, in US dollars (IQR)	2.0 (1.4–4.0)	3.0 (2.0–5.0)	1.8 (1.4–4.0)	1.2 (1.0–2.0)	3.0 (1.4–3.0)	0.00*

* Statistically significant

Abbreviations: hrHPV = high-risk human papillomavirus, IQR = interquartile range

Data from the site assessments are presented in Table 2. Site assessments were conducted at intervals averaging eight weeks. There were no statistical differences in site assessment metrics across the three sites included in the analysis. All three sites reported supply stockouts, two sites reported treatment delay due to lack of supplies, and two sites reported treatment delay due to provider factors. All sites had markedly fewer providers present than expected.

**Table 2:** Periodic assessments of four government hospitals providing cryotherapy in Migori County, Kenya (n = number of site assessments).

Finding	Macalder Sub-county Hospital n = 8*	Migori County Referral Hospital n = 5*	Karungu Sub-county Hospital n = 4*	p-value
Patients turned away per week	0 (0–1.5)	0 (0–0)	0 (0–0)	0.69
Unscheduled facility closure days in the past week	0 (0–0)	0 (0–0)	0 (0–0)	1.00
**Lack of supplies**				
At least one supply stocked out	4 (50%)	3 (60%)	1 (25%)	0.69
Treatment delay due to lack of supplies occurred at least once in the past week	3 (38%)	0 (0%)	1 (25%)	0.29
Cryotherapy machine lacked adequate gas in the past week	1 (13%)	0 (0%)	0 (0%)	1.00
**Lack of staff**				
Not enough staff to provide cryotherapy for all comers in the past week	1 (13%)	0 (0%)	0 (0%)	1.00
Treatment delay due to provider sick or busy occurred at least once in the past week	1 (13%)	2 (40%)	0 (0%)	0.39
Percentage of expected providers present per day	52% (47%-62%)	80% (57%-86%)	63% (53%-65%)	0.13

^*^ Data expressed as median (interquartile range) or frequency (%)

Surveys were conducted with five providers at Macalder, four at Ogwedhi, four at Migori, and three at Karungu, for a total of 16. One additional provider declined to participate, for a participation rate of 94%. Eighty-eight percent, 69%, and 88% of providers respectively agreed that concern for cancer, encouragement from family/friends, and outreach from study staff were factors motivating hrHPV+ patients to get treated. In terms of perceived patient facilitators of treatment, the options selected by providers are presented in Table 3. The top facilitator, according to providers, was providing treatment at no cost. The most important reported challenge in providing cryotherapy was workload from other patients, with the second being lack of working cryotherapy machine or gas. No providers endorsed daily challenges preventing provision of cryotherapy, but 20% endorsed them weekly, 33% two or three times a month, 33% once a month, and 13% never.

**Table 3:** Provider-identified facilitators, challenges for providers, and barriers for patients in provision of/access to treatment for hrHPV+ patients (n = 16).

Facilitator, challenge, or barrier	Providers selecting this option as most important (n, %)
**Facilitators of treatment**	
No cost for treatment	8 (50%)
Counseling from community health volunteers	3 (19%)
Understanding of where/how to get treatment	3 (19%)
Accessibility of treatment sites	1 (6%)
Efficiency of treatment visits	1 (6%)
**Challenges in providing cryotherapy**	
Workload from other patients	6 (40%)
Cryotherapy machine or gas broken/unavailable	5 (33%)
Not enough providers	3 (20%)
Inadequate training for providers	1 (7%)
**Barriers hrHPV+ women face in deciding to get treated**	
Lack of participant knowledge of hrHPV and cervical cancer	10 (67%)
Partner not supportive	2 (13%)
Family not supportive	2 (13%)
Disbelief in the need for treatment	1 (7%)
**Barriers hrHPV+ women face in accessing treatment once they have decided to get it**	
Financial barriers	7 (47%)
Partner not supportive	3 (20%)
Family not supportive	2 (13%)
Lack of transportation	2 (13%)
Distance to treatment site	1 (7%)

Abbreviations: hrHPV = high-risk human papillomavirus

In terms of barriers that hrHPV+ women face in deciding to get treated, the one most commonly selected was lack of knowledge of HPV and cervical cancer. For barriers that hrHPV+ women face in accessing treatment once they have decided to get it, financial barriers were most frequently selected. Lack of transportation and distance to treatment site–also chosen as top barriers–are intertwined with financial barriers, as evidenced by this quotation from a provider during an interview: “Let’s say they come from a far distance, they are not able to pay their transportation to the hospital.” Lack of partner support may also be linked to financial barriers, as women may rely on their partners for money for transportation. For example, one provider said, “When the partner is supportive, he will give you the fare to the facility.”

## Discussion

In the cervical cancer prevention program described in this study, there was a large attrition between notification of hrHPV+ status and presentation for treatment; in addition, a subset of women who presented for treatment were turned away for reasons other than contraindications to cryotherapy treatment. In the current work, we identified a number of systems-level barriers to help explain these high attrition rates.

Across all four treatment sites, the wait time for women seeking cryotherapy was substantial. While the transportation costs may seem inexpensive and the differences in costs small, they are not insignificant considering the average daily wage for an agricultural worker in Kenya is 11.40 USD [12] and Migori County is one of the poorest counties in Kenya. The fact that Migori County Referral Hospital had the lowest median transportation cost may have contributed to it having the highest treatment rate. This conjecture is supported by the emphasis in the provider survey data on financial and transportation barriers to treatment.

The data collected in the site assessments may underrepresent barriers to provision of cryotherapy. For example, at only two of 17 site assessments was it reported that women had been turned away from treatment in the past week. However, based on patient report, 15 total women were turned away during the treatment period, and it seems unlikely that all of them were turned away in just two weeks. In addition, at only one site assessment was inadequate gas for cryotherapy reported. In contrast, cryotherapy machine or gas broken/unavailable was the second most commonly selected top challenge in providing cryotherapy in the provider surveys. It may be the case that providers were more hesitant to reveal their centers’ limitations at the site assessments–which were quality check-ins asking them to quantify barriers to care specifically at their sites–than in the surveys, whose stated purpose was to elicit providers’ impressions about barriers to care more generally. Known challenges in the costs and availability of cryotherapy have led to the development and endorsement by the WHO of thermal ablation, an alternative treatment for cervical dysplasia that will potentially address some of these challenges [13, 14].

The barriers identified in this study overlap with barriers reported in other studies of cervical cancer prevention in sub-Saharan Africa. In previous work in Kenya, notable barriers included shortages of trained staff, inadequate space, lack of supplies, and long wait times [15]. Other studies in Tanzania, Botswana, and Uganda have reported similar barriers, with the addition of unavailability of screening results and rude treatment of patients by providers at health facilities [16–18].

A limitation of this study is that site assessments were not performed nearly as frequently as intended. There was only one site assessment for Ogwedhi, so this site was excluded from the site assessment analysis. The small number of assessments for each of the three other sites limited the power to detect differences between them. Another limitation of the study was that, since enumeration of patients turned away due to logistical/technical issues was based predominantly on patient report, it was likely an underestimate.

This study identified a number of potential areas for intervention to increase treatment rates in hrHPV+ women. Lack of understanding of HPV and cervical cancer was identified as a barrier to women deciding to get treated; this barrier could be lessened with a more extensive educational intervention than what was provided at the CHCs [19]. Although treatment was decentralized, there were still significant financial and logistical transportation hurdles for women to reach treatment sites. Providing mobile treatment or reimbursing patients for transportation costs might improve access to care. In terms of treatment site factors, the following were identified as potential barriers: long wait times, stockouts of supplies, outages of cryotherapy gas, and lack of sufficient providers for the patient workload. Stockouts of supplies and cryotherapy gas likely resulted both from shortage of funds and weaknesses in the supply chains to obtain these products. In terms of shortage of providers, it would be ideal to hire and train additional staff. The vast majority of these interventions to increase treatment rates for hrHPV+ women come with additional costs; as such, it is imperative to consider their relative values and sustainability before implementing them.

## Supporting information

S1 File Patient questionnaire. Administered to high-risk-HPV-positive women at visit for treatment.
(DOCX)

S2 File Site assessment form. Used to assess treatment sites during the treatment periods.
(DOCX)

S3 File Provider survey form. Used with providers from the treatment sites who were involved in counseling and/or providing treatment for high-risk-HPV-positive women.
(DOCX)

S1 Fig Flow diagram of study participants, beginning with all women who registered at community health campaigns. Abbreviations: hrHPV = high-risk human papillomavirus.
(TIFF)

S1 Table Characteristics of hrHPV positive women screened through a cervical cancer prevention program in Migori County, Kenya. (DOCX)

S1 Dataset De-identified patient data. (XLSX)

S2 Dataset De-identified site assessment data. (XLSX)

S3 Dataset De-identified provider survey data. (XLSX)

## References

[1] International Agency for Research on Cancer. Cancer fact sheets: Cervix uteri: Globocan; 2018 [cited 2020 April 2]. Available from: https://gco.iarc.fr/today/data/factsheets/cancers/23-Cervix-uteri-fact-sheet.pdf.

[2] Bruni L, Albero G, Serrano B, Mena M, Gómez D, Muñoz J, et al Human Papillomavirus and Related Diseases in Kenya. Summary Report. ICO/IARC Information Centre on HPV and Cancer (HPV Information Centre), 2019.

[3] Denny L, Kuhn L, Hu C-C, Tsai W-Y, Wright TC. Human Papillomavirus–Based Cervical Cancer Prevention: Long-term Results of a Randomized Screening Trial. JNCI: Journal of the National Cancer Institute. 2010;102:1557–67. 10.1093/jnci/djq342 20884893

[4] Sankaranarayanan R, Bhagwan N, Shastri S, Jayant K, Muwonge R, Budukh A, et al HPV screening for cervical cancer in rural India. New England Journal of Medicine. 2009;360:1385–94. 10.1056/NEJMoa0808516 19339719

[5] Geng EH, Bansberg DR, Musinguzi N, Emenyonu N, Bwana MB, Yiannoutsos CT, et al Understanding Reasons for and Outcomes of Patients Lost to Follow-Up in Antiretroviral Therapy Programs in Africa Through a Sampling-Based Approach. J Acquir Immune Defic Syndr. 2010;53:405–11. 10.1097/QAI.0b013e3181b843f0 19745753 PMC3606953

[6] Geng EH, Odeny TA, Lyamuya R, Nakiwogga-Muwanga A, Diero L, Bwana M, et al Retention in care and patient-reported reasons for undocumented transfer or stopping care among HIV-infected patients on antiretroviral therapy in Eastern Africa: Application of a sampling-based approach. Clinical Infectious Diseases. 2016;62:935–44. 10.1093/cid/civ1004 26679625 PMC4787603

[7] Page CM, Ibrahim S, Park LP, Huchko MJ. Patient factors affecting successful linkage to treatment in a cervical cancer prevention program in Kenya: A prospective cohort study. PLoS One. 2019;14(9):e0222750 10.1371/journal.pone.0222750 31532808 PMC6750649

[8] Huchko MJ, Kahn JG, Smith JS, Hiatt RA, Cohen CR, Bukusi E. Study protocol for a cluster-randomized trial to compare human papillomavirus based cervical cancer screening in community-health campaigns versus health facilities in western Kenya. BMC Cancer. 2017;17:1–12. 10.1186/s12885-016-3022-6 29207966 PMC5717798

[9] World Health Organization. Comprehensive cervical cancer prevention and control: a healthier future for girls and women. World Health Organization; 2013.

[10] Kenya Ministry of Health. Kenya National Cancer Screening Guidelines. Nairobi, 2018 [cited 2020 April 2]. Available from: http://www.health.go.ke/wp-content/uploads/2019/02/National-Cancer-Screening-Guidelines-2018.pdf.

[11] Santesso N, Schünemann H, Blumenthal P, De Vuyst H, Gage J, Garcia F, et al World Health Organization Guidelines: Use of cryotherapy for cervical intraepithelial neoplasia. International journal of gynaecology and obstetrics. 2012;118:97–102. 10.1016/j.ijgo.2012.01.029 22727415

[12] CEIC. Kenya Average Wage Earnings 2019 [cited 2019 March 11]. Available from: https://www.ceicdata.com/en/kenya/average-wage-earnings-by-sector-and-industry-international-standard-of-industrial-classification-rev-4/average-wage-earnings.

[13] World Health Organization. WHO guidelines for the use of thermal ablation for cervical pre-cancer lesions. World Health Organization; 2019. 31661202

[14] de Fouw M, Oosting RM, Rutgrink A, Dekkers OM, Peters AAW, Beltman JJ. A systematic review and meta-analysis of thermal coagulation compared with cryotherapy to treat precancerous cervical lesions in low- and middle-income countries. Int J Gynaecol Obstet. 2019;147(1):4–18. 10.1002/ijgo.12904 31273785

[15] Rosser JI, Hamisi S, Njoroge B, Huchko MJ. Barriers to Cervical Cancer Screening in Rural Kenya: Perspectives from a Provider Survey. J Community Health. 2015;40(4):756–61. 10.1007/s10900-015-9996-1 25677728 PMC8162879

[16] Matenge TG, Mash B. Barriers to accessing cervical cancer screening among HIV positive women in Kgatleng district, Botswana: A qualitative study. PLoS One. 2018;13(10):e0205425 10.1371/journal.pone.0205425 30356248 PMC6200249

[17] Bateman LB, Blakemore S, Koneru A, Mtesigwa T, McCree R, Lisovicz NF, et al Barriers and Facilitators to Cervical Cancer Screening, Diagnosis, Follow-Up Care and Treatment: Perspectives of Human Immunodeficiency Virus-Positive Women and Health Care Practitioners in Tanzania. Oncologist. 2019;24(1):69–75. 10.1634/theoncologist.2017-0444 29934410 PMC6324638

[18] Ndejjo R, Mukama T, Kiguli J, Musoke D. Knowledge, facilitators and barriers to cervical cancer screening among women in Uganda: a qualitative study. BMJ Open. 2017;7(6):e016282 10.1136/bmjopen-2017-016282 28606908 PMC5541520

[19] Rosser JI, Njoroge B, Huchko MJ. Changing knowledge, attitudes, and behaviors regarding cervical cancer screening: The effects of an educational intervention in rural Kenya. Patient Education and Counseling. 2015;98:884–9. 10.1016/j.pec.2015.03.017 25858634 PMC4437717

