# Supplementary material for: Systems-level barriers to treatment in a cervical cancer prevention program in Kenya: Several observational studies
Source: PLoS One. 2020 Jul 13;15(7):e0235264. doi: 10.1371/journal.pone.0235264 (PMC7357749; doi:10.1371/journal.pone.0235264)
Supplement: S1 File — Administered to high-risk-HPV-positive women at visit for treatment. (DOCX) [file pone.0235264.s001.docx]

| **PARTICIPANT TREATMENT DATA COLLECTION FORM** |
| --- |
| **SITE NAME: ___________________________**  **Hour Minute**  **Patient arrival time (reported) :**    **Hour Minute**  **Pretreatment start time: :**  **Day** **Month** **Year**    **Treatment Date:** / /   1. Provider name: _________________________________________________________________________   1b. Provider designation: Clinical Officer Nurse Other, Please specify _________________  1c. In which community were you tested? _____________________   1. What is the name of your village? _____________________________________   **First Name Middle Name Surname**   1. What is the name of your compound?     **First name Middle Name Surname**   1. What is your name?   **Day Month Year**   1. What is your date of birth? / /     5b. What is your age in years? _________________________________________  **If available, stick Barcode from brochure here. If not available write Barcode from notification SMS or**  **participant list**    BARCODE   1. Have you previously been treated for cervical cancer or precancer?: Yes **Go to Q6b** No **Go toQ7**   6b. Please describe previous treatment: Cryotherapy LEEP Chemotherapy Radiation  Other (specify) ________________________________________   1. Are you currently pregnant? Yes **DO NOT PROCEED WITH TREATMENT**, **GO TO Q22**   No **Go to Q7b**  Don't know **Go to Q7b**    7b**.** When (roughly in weeks) was your last menstrual period?  Less than 4 weeks ago **Proceed to treatment** Menopause **proceed to treatment.**  More than 4 weeks ago **Conduct pregnancy test** Other, specify **_____________________**  Don’t Know **Conduct pregnancy test**  7c. Urine pregnancy test result: Negative  **Proceed to treatment.**  Positive **DO NOT PROCEED WITH TREATMENT, GO TO Q22** |
| **PARTICIPANT TREATMENT** |
| **Hour Minute**  **Treatment start time: :**   1. Were you able to complete treatment today? Yes **Go to Q 8b** No **Go to Q22**   8b. What treatment did you receive?  Cryotherapy Biopsy  LEEP Referral  Other, specify __________________________ |
| **PARTICIPANT POST-TREATMENT EXPERIENCE** |
| **Hour Minute**  **Post-treatment start time: :**   1. Was today the first day you sought treatment? Yes **Go to Q10** No **Go to Q9b**     9b. Which treatment visit was this? __________________ (e.g.2, 3, 4 etc.)   1. Approximately how long ago did you receive your results?   Less than 1week  1 week - 1 month ago  1 month - 3 months ago  More than 3 months ago   1. How long does it take you to get to the clinic or Health Centre? Minutes 2. What type of transportation did you use?   Walked Motorbike/BodaBoda  Taxi/Matatu Boat  Other, specify:_______________________________   1. How much did the transportation cost (both ways)? **KSH**   13b. Was it difficult to find transportation here today? Yes No   1. Did you come with anyone today? Yes **Go to Q14b** No **Go to Q15**     14b. Who accompanied you?  Mother Sister Grandfather Friend  Father Brother Aunt Neighbor  Spouse Grandmother Uncle Other, specify: _________________   1. Did you tell anyone about your HPV results? Yes No 2. What is your primary occupation?     Farming Household worker Student  Business Healthcare worker Housewife  Fishing Factory worker Unemployed **Go to Q18**  Other, specify: _________________________________   1. Are you missing work today? Yes **Go to Q17b**  No **Go to Q17d**   17b. How many hours of work are you missing today? _______ Hrs.  17c. How much income are you losing today?  <1000 1001-2000 2001-3000 above 3001 Don’t Know  17d. Do you have childcare responsibilities at home? Yes No    17e. Is someone else caring for your children now that you are here? Yes No   1. Was the treatment procedure explained clearly? Yes No 2. Did you find the treatment uncomfortable or painful? Yes No 3. If a friend had a positive HPV test, would you recommend treatment for them? Yes No 4. Please add any additional comments or questions here: _________________________________________   ______________________________________________________________________________________  **Fill only if treatment was not completed**   1. If unable to complete treatment today, why? (**mark all that apply**)   menses  inadequate supplies/equipment at site  no trained staff member  changed my mind  not enough time to wait  was referred to another hospital  currently Pregnant  did not consent for pregnancy test  other, specify: ________________________________  **Hour Minute**  **Visit end time: :** |
|  |
