# Supplementary material for: Systems-level barriers to treatment in a cervical cancer prevention program in Kenya: Several observational studies
Source: PLoS One. 2020 Jul 13;15(7):e0235264. doi: 10.1371/journal.pone.0235264 (PMC7357749; doi:10.1371/journal.pone.0235264)
Supplement: S2 File — Used to assess treatment sites during the treatment periods. (DOCX) [file pone.0235264.s002.docx]

**TREATMENT SITE ASSESSMENT SURVEY**

**NOTE to interviewer: What type of visit is this? Please make sure you pick the right type of visit to be able to get the correct sets of questions for the visit answered. Multiple options allowed.**

- Baseline
- Bi-weekly

1 How many personnel work in the hospital on a typical day? (All departments)

2 How many medical staff work in the hospital on a typical day? (Doctors/COs, nurses, CHVs,

Counselors, etc.)

3 How many support staff work in the hospital on a typical day? (Cleaners, security, etc.)

4 How many Doctors are present in your unit today?

5 How many Doctors are expected to be present in your unit today?

6 How many COs are present in your unit today?

7 How many COs are expected to be present in your unit today?

8 How many nurses are present in your unit today?

9 How many nurses are expected to be present in your unit today?

10 How many community health workers/volunteers are present in your unit today?

11 How many community health workers/volunteers are expected to be present in your unit

today?

12 Apart from CHWs/CHVs how many trained counselors or peer educators are present in your unit

today?

13 Apart from CHWs/CHVs, ow many trained counselors or peer educators are expected to be

present in your unit today?

14 Are there any other support staff in this unit?

- Yes
- No

15 If so, which other personnel do you have in your unit? _____________________________

16 Are there other studies going on?

- Yes
- No

17 If so, which one(s)? __________________________________________________

**What counseling and education activities are being conducted at the facility**? Note: Please capture all counseling & education activities

19a HIV counseling

- Yes
- No
- Don’t know
- Declined to answer

19b Childhood Immunization counseling

- Yes
- No
- Don’t know
- Declined to answer

19c Family Planning Counseling

- Yes
- No
- Don’t know
- Declined to answer

19d Cervical Cancer Counseling

- Yes
- No
- Don’t know
- Declined to answer

19e Antenatal counseling

- Yes
- No
- Don’t know
- Declined to answer

19f Malaria counseling

- Yes
- No
- Don’t know
- Declined to answer

19g Non-communicable diseases counseling other than cervical cancer

- Yes
- No
- Don’t know
- Declined to answer

19h Other type of counseling and education specified __________________________________

**For each type of counseling/education activities indicated in the previous question, what days are these counseling and education activities conducted at the facility?** (Need to populate all that were checked in the previous question)

20a HIV counseling

- Mondays
- Tuesday
- Wednesday
- Thursday
- Friday
- Other

20b Childhood Immunization counseling

- Mondays
- Tuesday
- Wednesday
- Thursday
- Friday
- Other

20c Family Planning Counseling

- Mondays
- Tuesday
- Wednesday
- Thursday
- Friday
- Other

20d Cervical Cancer Counseling

- Mondays
- Tuesday
- Wednesday
- Thursday
- Friday
- Other

20e Antenatal counseling

- Mondays
- Tuesday
- Wednesday
- Thursday
- Friday
- Other

20f Malaria counseling

- Mondays
- Tuesday
- Wednesday
- Thursday
- Friday
- Other

20g Non-communicable diseases counseling other than cervical cancer

- Mondays
- Tuesday
- Wednesday
- Thursday
- Friday
- Other

20h Other type of counseling and education specified _________________________________

**For each type of counseling/education activities indicated in the previous question, how many times per day are these counseling and education activities conducted at the facility?** (Need to populate all that were checked in the previous question)

21a HIV counseling

- Once a day
- Twice a day
- Thrice a day
- Four or more times a day

21b Childhood Immunization counseling

- Once a day
- Twice a day
- Thrice a day
- Four or more times a day

21c Family Planning Counseling

- Once a day
- Twice a day
- Thrice a day
- Four or more times a day

21d Cervical Cancer Counseling

- Once a day
- Twice a day
- Thrice a day
- Four or more times a day

21e Antenatal counseling

- Once a day
- Twice a day
- Thrice a day
- Four or more times a day

21f Malaria counseling

- Once a day
- Twice a day
- Thrice a day
- Four or more times a day

21g Non-communicable diseases counseling other than cervical cancer

- Once a day
- Twice a day
- Thrice a day
- Four or more times a day

21h Other type of counseling and education specified _____________________________________

**For each type of counseling/education activities indicated in the previous question, who conducts the counseling and education activities being conducted at the facility?** (Need to populate all that were checked in the previous question)

22a HIV counseling

- Doctor
- Nurse
- Not certified CHWs
- Certified CHWs
- Counselors
- Other

22b Childhood Immunization counseling

- Doctor
- Nurse
- Not certified CHWs
- Certified CHWs
- Counselors
- Other

22c Family Planning Counseling

- Doctor
- Nurse
- Not certified CHWs
- Certified CHWs
- Counselors
- Other

22d Cervical Cancer Counseling

- Doctor
- Nurse
- Not certified CHWs
- Certified CHWs
- Counselors
- Other

22e Antenatal counseling

- Doctor
- Nurse
- Not certified CHWs
- Certified CHWs
- Counselors
- Other

22f Malaria counseling

- Doctor
- Nurse
- Not certified CHWs
- Certified CHWs
- Counselors
- Other

22g Non-communicable diseases counseling other than cervical cancer

- Doctor
- Nurse
- Not certified CHWs
- Certified CHWs
- Counselors
- Other

22h Other type of counseling and education specified ________________________

23 Prior to the study, was HPV or cervical cancer counseling provided for in this facility?

- - Yes
  - No
  - Don’t know
  - Declined to answer

24 Prior to the study, was HPV or cervical cancer treatment provided for in this facility?

- - Yes
  - No
  - Don’t know
  - Declined to answer

25 Are there any support groups/activities for patients who screen positive for HPV?

- - Yes
  - No
  - Don’t know
  - Declined to answer

26 Do patients ever need to be refered to another facility for services due to the lack of available

services at this hospital?

- - Yes
  - No
  - Don’t know
  - Declined to answer

27 If yes to previous question, how often?

- - Very often
  - Often
  - Less often/Rarely
  - Not at all
  - Other

28 What mode of transportation is most frequently used by the patients if they are referred to

another facility?

- - Walk
  - Taxi/Matatu
  - Motorbike/Boda Boda
  - Boat
  - Other

29 When there are emergencies in which patients are referred to another facility, how often do these patients travel to the referral facility by ambulance?

- Very often
- Often
- Less often/Rarely
- Not at all
- Other

30 How much time does it typically take for patients to reach the referral facility from this hospital by ambulance? __ __ : __ __ **[hh:mm]**

**Number of functional cryotherapy devices available today :**

31a Gloves (boxes)

31b Acetic acid

31c Sterile specula

31d Light source

31e Cotton wool

31f Clean mackintosh

31g Cryoprobes

31h Gas

31i Other device

31j Other functional device used for screening specified _______________________

Number of cryotherapy devices out of service or awaiting repair:

32a Light source

32b Cryoprobes

32c Gas

32d Other device

32e Other out of service device used for screening specified __________________________

**For each device that is not available/functional today, what is the reason? (Need to populate all devices not available)**

33a Light source

- Broken
- Missing
- Not available
- Someone else is using it
- Out of stock

33b Cryoprobes

- Broken
- Missing
- Not available
- Someone else is using it
- Out of stock

33c Gas

- Broken
- Missing
- Not available
- Someone else is using it
- Out of stock

33d Other device not available

- Broken
- Missing
- Not available
- Someone else is using it
- Out of stock

33e Other device used for screening not available specified ______________________________

**In the last week, how often was treatment provision delayed due to lack of the following supplies?**

34a Medicines

- Always
- Sometimes
- Not available at all

34b Gloves

- Always
- Sometimes
- Not available at all

34c Acetic acid

- Always
- Sometimes
- Not available at all

34d Sterile speculum

- Always
- Sometimes
- Not available at all

34e Light source

- Always
- Sometimes
- Not available at all

34f Cotton wool

- Always
- Sometimes
- Not available at all

34g Clean mackintosh

- Always
- Sometimes
- Not available at all

34h Autoclave

- Always
- Sometimes
- Not available at all

34i Cryoprobes

- Always
- Sometimes
- Not available at all

34j Gas

- Always
- Sometimes
- Not available at all

34k Other supplies

- Always
- Sometimes
- Not available at all

34l Other not available supplies used for screening specified _____________________________

**In the last 1 week, how often was treatment provision delayed due to factors related to providers?**

35a Providers were sick

- Very often
- Often
- Less often
- Rarely
- Not all

35b Providers had other responsibilities

- Very often
- Often
- Less often
- Rarely
- Not all

35c Lack of cryotherapy room available

- Very often
- Often
- Less often
- Rarely
- Not all

35d Other reason(s) __________________________________________

36 In the last week, how many days were there not enough trained staff to provide cryotherapy for

all patients who came to the hospital?

- - Never
  - 1-3 days
  - 4-6 days
  - Everyday

**In the last week, how often was treatment provision delayed due to factors related to patients?**

37a Patients came to the facility for other health issues and were seen in other department

- Very often
- Often
- Less often
- Rarely
- Not all

37b Mensturation

- Very often
- Often
- Less often
- Rarely
- Not all

37c Bad weather condition

- Very often
- Often
- Less often
- Rarely
- Not all

37d Patients had pain during treatment (and therefore delayed the procedure)

- Very often
- Often
- Less often
- Rarely
- Not all

37e Patients were concerned about or scared of the treatment

- Very often
- Often
- Less often
- Rarely
- Not all

37f Patients asked a lot of questions regarding the treatment procedure or HPV or cervical cancer

- Very often
  - Often
  - Less often
  - Rarely
  - Not all

37g Other reason(s) _______________________________________________________

38 Has the cryotherapy machine had adequate gas this week?

- - Yes
  - No
  - Don’t know
  - Declined to answer

39 If no, how many days has it not had gas?

40 On average, how much time do patients typically spend with a health worker during their visit (from time they walk-in to the time they walk-out clinic room, in Minutes)?

41 How many patients visit the health facility for outpatient services per week?

42 What is the number of Weekly client visit to the health facility for outpatient services?

43 How many patients were turned away from receiving treatment this Week?

44 How many days was the clinic closed due to weather or other unscheduled reasons last week?

- - Mondays
  - Tuesdays
  - Wednesdays
  - Thursdays
  - Fridays
  - other

46 What are the hours each day that the clinic is open?

- - Whole day 8am - 5pm
  - Morning hrs 8am - 12pm
  - Afternoon hrs 12pm - 5pm

47 In the last two weeks has the cryotherapy equipment been available to all patients including

those who are not participating in the study?

- - Yes to all
  - No, only for CCSP study participants
  - Don’t know

48 In the last two weeks how much were the patients being charged for cryotherapy or medical

supplies associated with their care?

- - Free
  - <1000
  - 1001-2000
  - 2001-3000
  - above 3001
  - Don’t Know

49 Are you trained on cryotherapy treatment?

- - Yes
  - No
  - Don’t know
  - Declined to answer

50 Do you feel confident in your ability to carry out these procedures?

- - Yes
  - No
  - Don’t know
  - Declined to answer

51 Are you receiving ongoing assistance/training/mentorship in performing cryotherapy?

- Yes
  - No
  - Don’t know
  - Declined to answer

52 How are patient records kept in your hospital? Paper/file records

- Electronic files
- Both paper and electronic filing
- Other

53 Are patient files retrieved to assess previous testing and treatment?

- Yes
- No
- Don’t know
- Declined to answer

54 How are patient records retrieved/reviewed at the time of visit?

- From Facility records
- From a file client brings with them
- From Study files
- Calls other Facility

55 How often does the cryotherapy get a maintenance checkup?

- Very often
  - Often
  - Less often/Rarely
  - Not at all
  - Other

56 Who is responsible for maintaining the cryotherapy machine?

- Study coordinator
- In-charge
- Nurses/Cos
- Others
