# Supplementary material for: Systems-level barriers to treatment in a cervical cancer prevention program in Kenya: Several observational studies
Source: PLoS One. 2020 Jul 13;15(7):e0235264. doi: 10.1371/journal.pone.0235264 (PMC7357749; doi:10.1371/journal.pone.0235264)
Supplement: S3 File — Used with providers from the treatment sites who were involved in counseling and/or providing treatment for high-risk-HPV-positive women. (DOCX) [file pone.0235264.s003.docx]

**COMMUNITY BASED HPV TESTING – Treatment Provider Survey**

| **Participant ID: ______________________**    **Date: ____ / ___ /__________**  **dd mm yyyy**  **Interviewer: ________________________________**  **Community Screening Site: ___________________** |
| --- |
| **Interviewer: “Thank you for taking the time to meet with me today. My name is ____________ and I’d like to ask you a few questions to help us understand how to improve cervical cancer treatment in your community. Feel free not to answer any question that makes you uncomfortable.** |

1. **DEMOGRAPHIC CHARACTERISTICS**

**First I’d like to ask you some simple demographic questions.**

1. What is your age?
2. What is your gender?

__ Male __ Female

1. What is your role at the clinic?

__ Clinical officer __ Nurse

__ Administrator/receptionist __ Other: _______________________

__ Community health volunteer

1. How long have you been working at this clinic?

__ 0-2 months __ 6-12 months

__ 2-6 months __ >12 months

1. How many total years of experience do you have in healthcare?

__ <1 year __ 2-5 years

__ 1-2 years __ >5 years

1. How many total years of experience do you have in women’s health in particular (e.g. family planning, antenatal care, cervical cancer screening)?

__ <1 year __ 2-5 years

__ 1-2 years __ >5 years

1. What proportion of your time do you spend on cervical cancer-related activities?

__ <25% __ 75-100%

__ 25-50% __ All my time

__ 50-75%

1. What is your role with cervical cancer screening or treatment? *(can choose multiple options)*

__ Screening __ Providing treatment *(🡪 continue survey*)

__ Maintaining supplies __ Counseling *(🡪 continue survey)*

__ Administrative __ Other

__ Outreach __ None

1. **QUESTIONS FOR BOTH PROVIDERS AND COUNSELORS**
2. What do you think motivates patients to get treated if they’re HPV positive? *(can choose multiple options)*

__ Concern for cancer __ Outreach from study staff

__ Encouragement from family/friends

1. What do you think facilitates treatment of HPV-positive patients in your facility? *(can choose multiple options)*

__ Counseling from community health volunteers

__ Understanding of where/how to get treatment

__ Accessibility of treatment sites (not too far, roads okay)

__ Assistance with transportation to treatment sites

__ Friendliness/professional nature of providers

__ Efficiency of treatment visits (not too long waiting before or during the visits)

__ No cost for treatment

__ Treatment is completed in a single visit

__ Understanding of treatment techniques

__ Hearing peers’ experiences

__ Family support

__ Don’t know

__ Other: _________________________________

1. What do you think is the #1 thing that facilitates treatment of HPV-positive patients in your facility?

__ Counseling from community health volunteers

__ Understanding of where/how to get treatment

__ Accessibility of treatment sites (not too far, roads okay)

__ Assistance with transportation to treatment sites

__ Friendliness/professional nature of providers

__ Efficiency of treatment visits (not too long waiting before or during the visits)

__ No cost for treatment

__ Treatment is completed in a single visit

__ Understanding of treatment techniques

__ Hearing peers’ experiences

__ Family support

__ Don’t know

__ Other: _________________________________

1. **QUESTIONS FOR PROVIDERS**
2. Do you have training for cervical cancer prevention treatment (cryotherapy or LEEP)?

__ Yes __ No *(🡪 skip to question 17)*

1. What type of treatment are you trained for? *(can choose multiple options)*

__ LEEP __ Hysterectomy

__ Cold-knife cone __ Other

__ Cryotherapy

1. What type of training have you had for treatment?

__ On the job __ Off-site courses

__ In school __ Other

1. How long was the training?

__ 1 day __ >5 days

__ 2-5 days

1. Did you feel the training was adequate?

__ Definitely __ No

__ Somewhat

1. How comfortable do you feel as a provider of cryotherapy?

__ Very comfortable __ Uncomfortable

__ Somewhat comfortable

1. Why did you get trained in cryotherapy?

__ Assigned to do it __ Interested in learning new skills

__ Wanted financial incentive __ Wanted certificate

__ Felt that it’s an important service for patients

1. How long does a treatment visit take, from the time you arrive at the hospital until you leave?

__ 1-15 min __ 46-60 min

__ 16-30 min __ >60 min

__ 31-45 min

1. How often has a patient had a complication after cryotherapy performed by you?

__ Never *(🡪 skip to question 22)* __ Greater than 5 times

__ 1-5 times

1. What type(s) of complication(s)? *(can choose multiple options)*

__ Infection __ Heavy bleeding

__ Severe pain __ Other

1. Has a patient ever declined a pelvic exam due to your gender?

__ Yes, often __ No

__ Yes, sometimes

1. Has a patient ever declined treatment after counseling?

__ Yes __ No *(🡪 skip to question 25)*

1. Why did they decline? *(can choose multiple options)*

__ Fear of discomfort __ Fear of immodesty

__ Not enough time __ Unknown reason

1. What challenges do you face in providing cryotherapy? *(can choose multiple options)*

__ Cryotherapy machine or gas broken/unavailable

__ Lack of gloves, acetic acid, etc.

__ Lack of support staff __ Inadequate training for myself

__ Patient fear __ Patient discomfort /pain with procedure

__ Not enough providers __ Difficult to counsel patients on sensitive topic

__ Workload from other patients __ None *(🡪 done with survey)*

1. What is the #1 challenge you face in providing cryotherapy?

__ Cryotherapy machine or gas broken/unavailable

__ Lack of gloves, acetic acid, etc.

__ Lack of support staff __ Inadequate training for myself

__ Patient fear __ Patient discomfort /pain with procedure

__ Not enough providers __ Difficult to counsel patients on sensitive topic

__ Workload from other patients __ None

1. How often do you face challenges that prevent you from performing cryotherapy?

__ Never __ Weekly

__ Once a month __ Almost every day

__ 2-3 times a month

1. **QUESTIONS FOR COUNSELORS**
2. What type of training have you had for counseling? *(can choose multiple)*

__ On the job __ Off site courses

__ In school __ Other

__ None (🡪 skip to question 31)

1. How long was the training?

__ 1 day __ 2-5 days

__ >5 days

1. Did you feel the training was adequate?

__ Definitely __ Somewhat

__ No

1. What challenges do you face in counseling patients to get treated if they’re HPV positive? *(can choose multiple)*

__ Lack of personal knowledge of HPV and cervical cancer

__ Lack of training in counseling methods

__ Not enough time to fully discuss results and treatment

__ Lack of helpful visual aids for counseling

__ Low levels of participant health knowledge

__ Lack of funds for transport for home visits

1. What barriers do you think HPV-positive women face in deciding to get treatment?

__ Lack of participant knowledge of HPV and cervical cancer

__ Disbelief in the need for treatment

__ Lack of confidence/belief in their HPV result

__ Distrust of the medical system

__ Partner not supportive

__ Family not supportive

1. What do you think is the #1 barrier HPV-positive women face in deciding to get treatment?

__ Lack of participant knowledge of HPV and cervical cancer

__ Disbelief in the need for treatment

__ Lack of confidence/belief in their HPV result

__ Distrust of the medical system

__ Partner not supportive

__ Family not supportive

1. What barriers do you think HPV-positive women face in accessing treatment after they have made the decision to get it?

__ Lack of transportation

__ Work/childcare barriers (unable to get time off)

__ Financial barriers

__ Distance to treatment site

__ Family not supportive

__ Partner not supportive

1. What do you think is the #1 barrier HPV-positive women face in accessing treatment after they have made the decision to get it?

__ Lack of transportation

__ Work/childcare barriers (unable to get time off)

__ Financial barriers

__ Distance to treatment site

__ Family not supportive

__ Partner not supportive
