# Supplementary figures and images for: Systems-level barriers to treatment in a cervical cancer prevention program in Kenya: Several observational studies
Source: PLoS One. 2020 Jul 13;15(7):e0235264. doi: 10.1371/journal.pone.0235264 (PMC7357749; doi:10.1371/journal.pone.0235264)

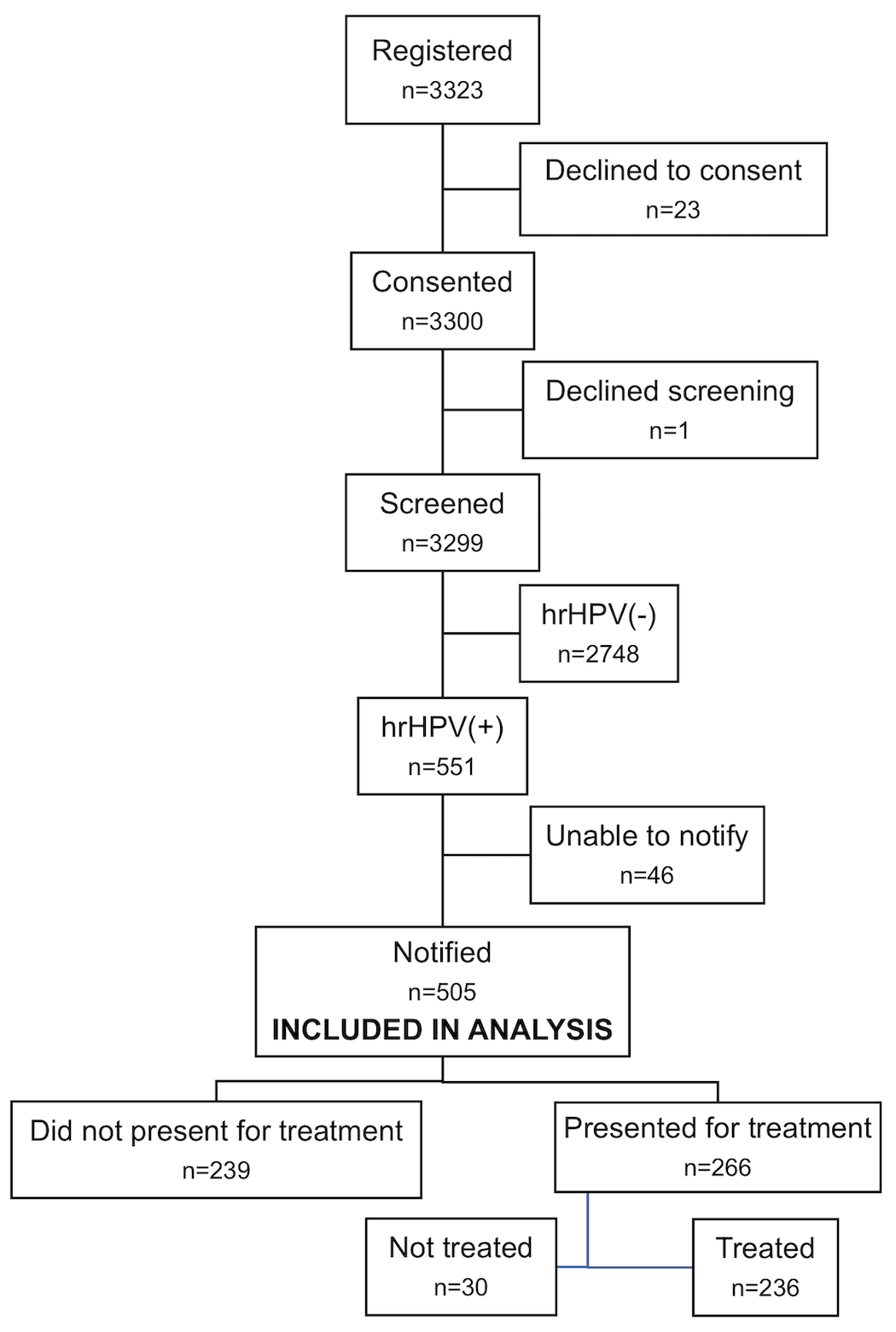

Supplement: S1 Fig — Abbreviations: hrHPV = high-risk human papillomavirus. (TIFF) [file pone.0235264.s004.tiff]
