# Supplementary material for: Systems-level barriers to treatment in a cervical cancer prevention program in Kenya: Several observational studies
Source: PLoS One. 2020 Jul 13;15(7):e0235264. doi: 10.1371/journal.pone.0235264 (PMC7357749; doi:10.1371/journal.pone.0235264)
Supplement: S1 Table — (DOCX) [file pone.0235264.s005.docx]

**S1 Table. Characteristics of hrHPV positive women screened through a cervical cancer prevention program in Migori County, Kenya (N=505)**

| **Patient factor** | **Category** | **Median (interquartile range) or frequency (%)** |
| --- | --- | --- |
| Age (years) | -- | 33 (27-42) |
| Total children | 0-2 | 134 (27%) |
|  | 3-4 | 166 (33%) |
|  | 5+ | 205 (41%) |
| Children under age 13 | 0-2 | 306 (61%) |
|  | 3-4 | 171 (34%) |
|  | 5+ | 28 (6%) |
| Education level | Primary school or less | 428 (85%) |
|  | At least some secondary | 77 (15%) |
| Relationship status | Not partnered | 132 (27%) |
|  | Partnered | 366 (73%) |
| Uses a cellphone | No | 87 (17%) |
|  | Yes | 418 (83%) |
| Has a personal cellphone | No | 135 (27%) |
|  | Yes | 365 (73%) |
| Works outside the home | No | 214 (42%) |
|  | Yes | 291 (58%) |
| Used paid transportation to get to the CHC | No | 494 (98%) |
|  | Yes | 11 (2%) |
| Frequency of depressive symptoms | Never | 140 (28%) |
|  | Some days | 227 (45%) |
|  | Most or almost every day | 138 (27%) |
| Ever diagnosed with depression | No | 467 (92%) |
|  | Yes | 38 (8%) |
| Distance to treatment site (kilometers) | -- | 8 (5-12) |

Abbreviations: CHC = community health campaign, hrHPV = high-risk human papillomavirus
